# Effects of Voluntary Alcohol Intake on Risk Preference and Behavioral Flexibility during Rat Adolescence

**DOI:** 10.1371/journal.pone.0100697

**Published:** 2014-07-09

**Authors:** Matthew S. McMurray, Leslie R. Amodeo, Jamie D. Roitman

**Affiliations:** 1 Department of Psychology, University of Illinois at Chicago, Chicago, Illinois, United States of America; 2 Laboratory of Integrative Neuroscience, University of Illinois at Chicago, Chicago, Illinois, United States of America; University of Lethbridge, Canada

## Abstract

Alcohol use is common in adolescence, with a large portion of intake occurring during episodes of binging. This pattern of alcohol consumption coincides with a critical period for neurocognitive development and may impact decision-making and reward processing. Prior studies have demonstrated alterations in adult decision-making following adolescent usage, but it remains to be seen if these alterations exist in adolescence, or are latent until adulthood. Here, using a translational model of voluntary binge alcohol consumption in adolescents, we assess the impact of alcohol intake on risk preference and behavioral flexibility during adolescence. During adolescence (postnatal day 30–50), rats were given 1-hour access to either a 10% alcohol gelatin mixture (EtOH) or a calorie equivalent gelatin (Control) at the onset of the dark cycle. EtOH consuming rats were classified as either High or Low consumers based on intake levels. Adolescent rats underwent behavioral testing once a day, with one group performing a risk preference task, and a second group performing a reversal-learning task during the 20-day period of gelatin access. EtOH-High rats showed increases in risk preference compared to Control rats, but not EtOH-Low animals. However, adolescent rats did a poor job of matching their behavior to optimize outcomes, suggesting that adolescents may adopt a response bias. In addition, adolescent ethanol exposure did not affect the animals' ability to flexibly adapt behavior to changing reward contingencies during reversal learning. These data support the view that adolescent alcohol consumption can have short-term detrimental effects on risk-taking when examined during adolescence, which does not seem to be attributable to an inability to flexibly encode reward contingencies on behavioral responses.

## Introduction

Adolescence is a developmental transition from childhood to adulthood when one expects to achieve a level of independence and stability. During this period, decisions often become increasingly rebellious and consequently risky, including experimentation with alcohol and illicit drugs. Recent evidence has shown that approximately 40–50% of 18–22 year olds in the U.S. report having consumed alcohol in the past 30 days [1]. Further, adolescents show a higher rate of binge drinking (25.6%) than the general population of adults (15.2%) [2]. Adolescents also typically favor sweetened/flavored alcohol solutions [3] and show a disproportionate consumption of “jello shots” compared to adults, representing a substantial proportion of their total alcohol intake [4]. Animal models of adolescent alcohol consumption demonstrate a repertoire of behaviors similar to adolescent humans [5], [6], including the preferential consumption of alcohol in gelatin form on a g/kg body weight basis [7].

Aside from changes in drug-seeking, adolescence is also a critical period for brain development, and is accompanied by the maturation of neurocognitive processes that underlie inhibitory control, decision-making, and reward processing [8], [9]. Tasks of selective attention, working-memory and problem-solving are all correlated with fronto-cortical synaptic pruning and myelination throughout adolescent development [10]. In general, divergences in the developmental trajectory of adolescent prefrontal cortex and subcortical systems might explain differences in adolescent behavior compared to their older and younger counterparts [11], [12]. For example, adolescents demonstrate reduced right anterior cingulate, left orbitofrontal, and left ventrolateral prefrontal cortex activation during a decision-making task involving risky choices [13] and stronger ventral striatum and orbitofrontal cortex activation during a Stoplight driving game in which they took more risks [14], all relative to adults. These studies support the notion that altered patterns of frontal and striatal activation in the adolescent brain is related to differences in cognitive performance and reward processing compared to adults [10].

Chronic alcohol use has been shown to cause detrimental effects on cognitive processing and prefrontal cortex functioning in adolescent animals [15], [16], [17], [18]. Studies have shown that the forebrain regions in adolescent rats are especially sensitive to alcohol-induced neurodegeneration and inhibition of neurogenesis [19], [20]. In particular, damage to associated frontal cortical olfactory regions and anterior portions of the piriform and perirhinal cortices after binge drinking are unique to the adolescent population and are not expressed in adults [21]. Perturbations of the normal developmental trajectory of these regions could also have far reaching effects on decision-making and behavior. Following a period of abstinence after continuous voluntary alcohol exposure in adolescence, adult rats displayed choice biases towards large, risky rewards over small, certain rewards, a task known to involve these brain regions [7], [22], [23]. This effect was seen both 3 weeks and 3 months after discontinuation of alcohol access. Adults also show deficits in behavioral flexibility following adolescent ethanol exposure [16], [24], [25], [26]. Together, these studies suggest that alcohol can be neurotoxic to the adolescent brain, disrupting cortical remodeling, resulting in maladaptive behavior.

While voluntary consumption has been shown to have consequences for subsequent decision-making in adulthood [7], its immediate impact during adolescence has not been shown. Alterations in decision-making could either result from adolescent drug usage or could pre-exist drug exposure. In an effort to address this question, here we have examined the effects of alcohol on two types of decision-making tasks in adolescents: 1) when risk is a factor (risk preference); and 2) in response to changing reward contingencies (behavioral flexibility). We hypothesized that rats given access to alcohol during adolescence will show stronger risk preference and an increase in perseverative behavior when testing during the period of administration. Aside from illustrating the immediate impairments following adolescent alcohol intake, differences in behavior between the groups may suggest that alcohol ingestion in adolescence drives deficits in decision-making, rather than pre-existing deficits in decision-making driving increased alcohol consumption. Our data support the view that alcohol consumption during adolescence can have a detrimental effect on risk-taking behavior when examined in adolescence, which does not seem to be attributable to an inability to flexibly encode changing reward contingencies.

## Materials and Methods

### Subjects

34 male Sprague-Dawley rats (Charles River Laboratory, Portage, MI) arrived in the laboratory on postnatal day 22 (P22). Rats were housed in groups of 4 (2 control and 2 alcohol, detailed below) in large polycarbonate cages (56×34×22 cm) and provided lab chow (LabDiet 5012, Richmond, IN) and water *ad libitum*. The colony was maintained on a 12∶12 light/dark cycle (7:00AM–7:00PM) with behavioral testing conducted in a separate experimental room during the light phase of the cycle. Animals were treated in accordance with the guidelines put forth by the National Institutes of Health and under the approval of the Animal Care Committee of the University of Illinois at Chicago.

### Adolescent Ethanol Access

**Figure 1:**
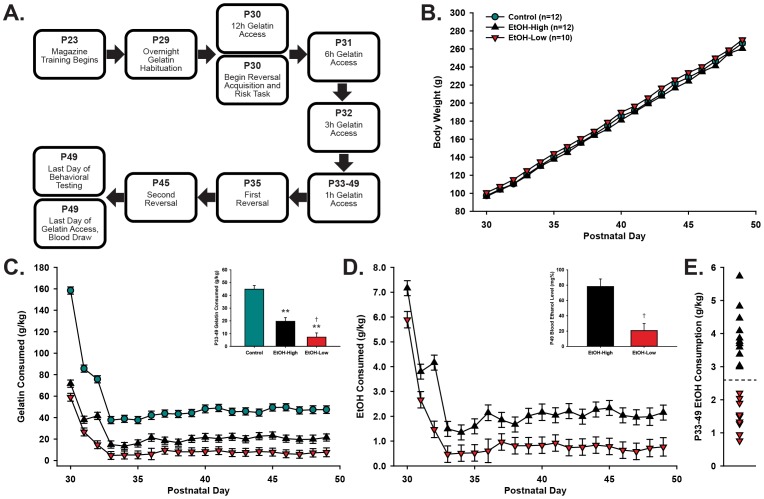
An overview of the experiment flow and resulting intake levels. Animals were given voluntary access to gelatin (control or 10% ethanol) and underwent behavioral testing throughout adolescence (A). There were no differences in weight gain across development (B), despite consistent differences in consumption between groups across adolescence (C, inset). Ethanol consumption (g/kg) was stable across 1 h access days (D), resulting in differential BELs among High and Low EtOH consumers (D, inset). High and Low consumers were grouped according to a median split (dashed line) of their average intake on 1 hour access days (E). Note: ** denotes difference from Control (p≤0.001); † denotes difference from EtOH-High (p≤0.05).

Alcohol was presented in a gel comprised of distilled water, 2.5% Knox^©^ gelatin (Kraft Foods, Northfield, IL), 10% Polycose (Abbot Laboratories, Columbus, Ohio), and 10% EtOH by weight [27]. A non-alcoholic control gelatin was presented to control animals throughout exposure. To account for the calorie discrepancy between Ethanol and Control gelatin, the percentage of polycose was increased in the Control diet, such that the diets were calorie-matched by volume. Gelatin was provided using a modified “drinking-in-the-dark-multiple-scheduled-access” paradigm [27], [28], [29], which permitted access at the onset of the dark cycle for 12 hr on P29 and P30, 6 hr on P31, 3 hr on P32, and 1 hr daily from P33 to P49 (Figure 1A). During the daily access period to gelatin, mesh dividers were inserted into the home cage to allow animals social contact, while maintaining separate access to gelatin for accurate measurement of consumption.

On the pre-exposure day (P29), all animals were given 12 hr overnight access to control gelatin to reduce neophobia during the first EtOH gelatin access period. Of the 34 animals tested, all animals consumed at least 5 g of gelatin during this period. Animals were then matched by weight and split into two groups: Ethanol (ad lib EtOH gelatin, n = 22) and Control (ad lib gelatin, n = 12). EtOH-treated rats demonstrated a natural split of low and high consumers when intake was averaged over 1 hr access days (P33-49, Figure 1E). Thus, to better represent the data, we divided EtOH rats into two groups, EtOH-High (n = 12) and EtOH-Low (n = 10), using a median split of 2.6 g gelatin intake. After each daily access period, jar weights were recorded and gelatin consumption was calculated in g/kg for each animal, using the individual body weights measured that day. On the last night of gelatin access (P49), blood was taken via tail nick to measure blood ethanol levels (BELs, detailed below). Behavioral testing took place on days P30–49, no sooner than 5 hours after termination of alcohol access.

### Testing Apparatus

Behavioral testing was conducted in four identical mouse-sized operant chambers (30.5 cm×24.0 cm×29.0 cm) housed inside sound-attenuating boxes (Med Associates, St. Albans, VT). Nosepoke ports (2.5 cm diameter) with three colored lights were located to the left and right of a retractable sipper tube, which could be accessed through a hole located between the nosepoking devices. This hole and the nosepoke ports were equipped with infrared beams to detect head-entries. A lickometer circuit was used to monitor licking behavior during the test sessions.

### Experiment 1: Risk Task

The risk task was adapted from previously published work from our lab [30]. Prior to initiating the risk task, 19 adolescent rats (7 Control, 7 EtOH-High, 5 EtOH-Low) underwent magazine training on P23–24, in which nosepokes into either of the two illuminated nosepoke ports would result in access to liquid reward via a sipper tube (50% Ensure liquid diet solution diluted in tap water (Milk Chocolate; Abbot Laboratories, Columbus, OH). All rats successfully learned to nosepoke in either port for reward in this time period. Following magazine training, all animals underwent a modified version of the variable risk task (full task detailed below), in which the probability of risky reward was fixed at 100% (P25–29). Only data from the final day of 100% probability (P29) was analyzed.

Risk preference assessment took place during daily test sessions from P30–49, with each behavioral session consisting of two blocks of trials: ‘forced response’ and ‘free choice’. The ‘forced response’ block consisted of the first 8 trials, in which only one of the two possible nosepoke ports were illuminated on each trial. For each subject, one port was designated as ‘certain’ and the other ‘risky’, with the designation counterbalanced across rats. The first trial began with the illumination of the cue light in the certain port to indicate that it was active. Once poked, the cue light was extinguished and the sipper tube inserted into the chamber. The rat was then allowed 5 licks of Ensure. Once the five licks were completed, the sipper retracted and a 5 or 10 s variable interval separated consecutive trials, with the house light extinguished during this period. On the second trial, only the risky port became active and the associated cue light illuminated. When poked, the cue light was extinguished, and either the sipper tube extended into the chamber for 15 licks of Ensure or the house light extinguished to indicate a failed trial, and the next trial began after 5–10 s. The probability of reward delivery following uncertain nosepokes was 25%, 50%, or 75%, which was maintained throughout the entire daily session and presented pseudo-randomly across sessions, such that the same probability was never presented twice in a row. Rats received 7 sessions on each probability. For any given response in the risky port, reward delivery was determined randomly, with replacement, according to the session's reward schedule to ensure unpredictable outcomes across a series of trials (i.e. on 25%, 1 out of every 4 risky choices was reinforced). Rats were allowed 15 licks of Ensure for rewarded risky trials, and zero for unrewarded. For the remaining ‘forced’ trials, each nosepoke port was illuminated in alternation. The ‘forced response’ block ensured that subjects had equal experience with both operant options and their associated outcomes at the start of each session. Although rats received uncertain outcomes on half of the trials, they did not have to decide which behavioral response to make (only one port was illuminated and active).

**Figure 2:**
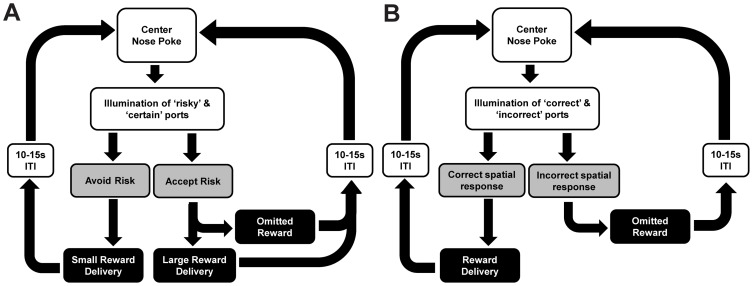
The sequence of trial events on the Risk Task (A) and Reversal Learning Task (B).

Immediately following the initial block of 8 forced trials, subjects had up to 150 additional ‘free choice’ trials (Figure 2A), although no animals reached this maximum. On each trial, the illumination of cue lights in both nosepoke ports indicated that both ports were armed. Both ports remained available until a port's infrared beam was activated by the rat. Upon the behavioral response, both cue lights extinguished and the sipper tube extended into the chamber when appropriate (e.g. certain and risky success). For certain choices, the rat received 5 licks of Ensure, and for risky choices he received either zero or 15 licks, assigned randomly with replacement, so that subjects could not anticipate or track outcome across the session. Again, the probability of risky success varied between 25%, 50%, or 75%. It is important to note that in the free choice block, rats had the option to receive 5 licks of Ensure on every trial. Receiving a larger reward following a risky choice was therefore a relative gain for those trials, while reward omission was a relative loss compared with the certain outcome. Risk preference was quantified as the proportion of risky port choices during the free choice block.

### Experiment 2: Reversal Learning Task

15 Adolescent rats were magazine trained from P23–29 in an identical manner as in Experiment 1 and began the reversal learning paradigm on P30. In this task, a nosepoke in the central port was required to initiate the trial, resulting in the illumination of two spatially distinct ports. During the acquisition phase of this task, a correct nosepoke in the rewarded port resulted in the simultaneous extinction of the port lights and extension of the sipper tube for 10 licks of Ensure. Incorrect nosepokes in the opposing port also caused the extinction of the port lights, but resulted in the omission of the reward (no spout extension). At the end of each trial, the house light was turned off for a varying inter-trial interval of 10 or 15 s. Following this, the house light turned on, cueing the rat to start the next trial with a nosepoke at the center port. The rewarded and omitted sides were counterbalanced across subjects. The session ended after 30 trials or 1 h.

Following acquisition, the consequences of responses to each location were reversed on P35 and again on P45. Acquisition criterion was attained when a rat achieved 70% correct for two consecutive sessions. Because all rats underwent reversals on the same developmental days (P35 and 45), rats that reached criterion before the reversal day were run daily on a maintenance task, in which they were placed the chamber and given the maximum amount of reward possible on a test day, 300 licks of ensure. All rats were given a retention test the day prior to reversal. This was conducted to ensure there were no differences in retaining the initially learned discrimination.

An analysis of errors during reversal learning was conducted to determine whether there were any differences between EtOH and control rats in the ability to initially inhibit the previously rewarded choice (perseverative error) and the ability to maintain the new choice pattern after demonstrating a bias for the new rewarded contingencies (regressive error) [31]. To determine the number of perseverative errors, trials were separated into consecutive blocks of three trials for a total of 10 blocks per session. When a rat chose the previously correct spatial location on the majority of the trials in that block, it was scored as a ‘perseverative error’. The first block composed of two or more consecutive nosepokes on the new correct spatial location was considered a successful block, and subsequent blocks composed of majority errors were scored as ‘regressive errors’. Each daily session was analyzed separately.

### Blood Ethanol Level Assessment

Blood ethanol levels (BEL) were measured for all subjects (both EtOH and control groups) on P49 immediately following the last hour-long gelatin access period. Tails were cleaned and sterile single blade razors were used to make a small tail-tip nick for blood collection. Blood samples were stored in heparin at −60°C until measurement. Tail blood (6 µl) and standards (6 µl; 0–200 mg%) were mixed with 375 ml of distilled water and 0.5 g NaCl in 12×75 mm borosilicate glass culture tubes. The tubes were capped and then heated to 55°C for 10-min in a water bath, at which point 1.5 ml of headspace gas was removed with a plastic 3.0 ml syringe and injected directly into an SRI 8610C gas chromatograph (Torrance, CA) equipped with an external syringe adapter and 1.0 ml external loading loop. The oven temperature was isothermal at 140°C and contained a Hayesep D column and a flame ionization detector. Hydrogen gas, carrier gas (also hydrogen), and internal air generator flow rates were 13.3, 25, and 250 ml/min respectively. Peak retention time was 2 min and the areas under the curve were analyzed with SRI PeakSimple software for Windows running on a Dell Inspiron 3500 laptop computer.

### Statistical Analysis

Body weight and gelatin consumption (g/kg) were analyzed using two way (group × day) repeated measures ANOVAs followed by posthoc tukey tests where appropriate. For consumption analyses, only days with one hour access were included in the statistical model. BELs were compared using a T-test. Risk preference was assessed using a three-way (group × probability × session number) repeated measures mixed model, followed by contrasts to determine specific differences. All contrasts in this model were corrected for multiple comparisons using the FDR method, and effect sizes for each significant contrast were calculated using Cohen's d. The number of trials completed and number of licks earned were analyzed using separate two-way repeated measures ANOVAs followed by posthoc tukey tests where appropriate, with effects sizes for significant effects calculated using η^2^ (Eta squared). Reversal data was analyzed using two-way (group × day) repeated measures ANOVA followed by tukey tests. Alpha levels were set a 0.05 for all tests. Means and standard errors for each data type are presented in figures. All statistical analyses were completed using SigmaPlot (v12.5) or SAS (v9.2).

## Results

### Voluntary adolescent ethanol consumption and body weight

Adolescent rats were provided access to gelatin (10% EtOH or Control) during a limited period across P30–49. The mean body weights of adolescent rats (Figure 1B) increased progressively across this period [*F*(19,583) = 1895.41, *p*<0.001], but did not vary by treatment group [F(2,583) = 0.60, p = 0.55]. Starting on P30, rats were given 12 h ad lib access to the gelatin, followed by 6 h, 3 h, and then 1 h access on subsequent days. In the initial tapering of gelatin access period (P30–32), there was an expected overall decline in intake (Figure 1C), followed by a stable level of consumption (g/kg) across the remainder of the adolescent period (P33–49). Across days with one hour access periods, Control rats consumed significantly more gelatin [F(2,474) = 40.03, p<0.001] than both EtOH-High (p≤0.001) and EtOH-Low (p≤0.001) consuming rats, and EtOH-High rats consumed significantly more than EtOH-Low rats (p = 0.022). Daily EtOH intake and average intake over the 1 h access days (P33–49) is shown in Figures 1D and E, respectively. After the final day of ethanol access, EtOH-High rats had significantly elevated BELs (78.3±9.8 mg%) compared EtOH-Low (20.6±9.4 mg%, t = 3.991, p≤0.01).

### Experiment 1: Effects of adolescent alcohol exposure on risk preference

**Figure 3:**
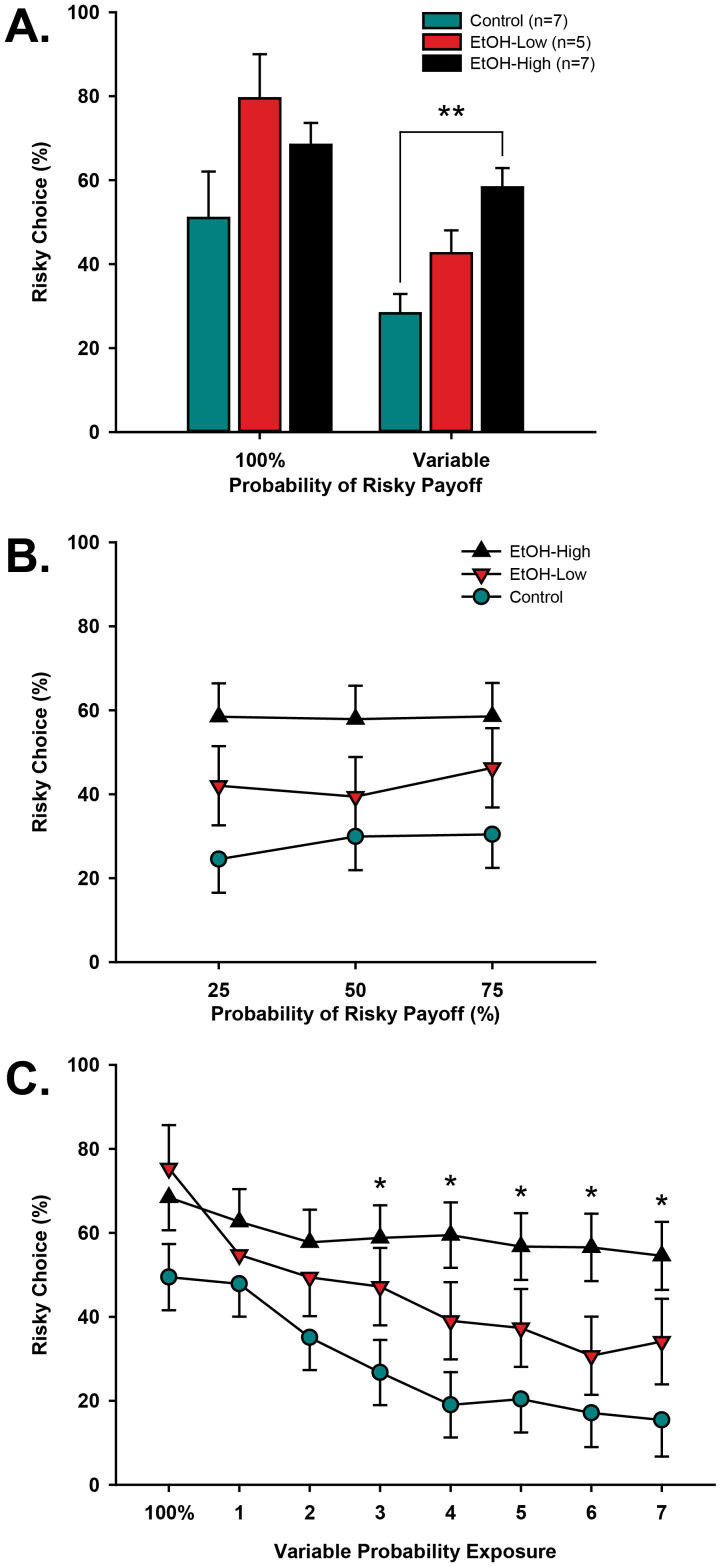
The effects of adolescent alcohol on adolescent risk preference. No difference between groups existed on 100% rewarded trials; however, when the probability became variable, EtOH-High animals chose the risky option more often than control animals (A), and regardless of the probability of success (B). Both Control and EtOH-Low animals showed decreasing risk preference over continued exposure to variable risk, while EtOH-High animals showed no decrease in risk preference over time (C). The order of variable probabilities was randomly selected for each animal, thus “Variable Probability Exposure” refers to the average performance across probabilities (e.g. 1 =  average performance across first exposures to 25%, 50%, and 75% probabilities). Note: * denotes difference from Control (*p≤0.05, **p<0.01).

In the risk task (Figure 2A), risk preference was measured as the proportion of nosepokes into the risky port over all free choice trials in the session. All risk preference data were analyzed using a single three-way repeated measures mixed model (group × probability × session number), which showed significant main effects of Group [F(2,305) = 4.36, p = 0.014] and Session Number [F(7,305) = 22.35, p≤0.0001], and a significant Group × Session interaction [F(14,305) = 3.38, p p≤0.0001]. There was no effect of probability on risk preference [F(3,305) = 0.91, p = 0.41], suggesting that adolescent animals, regardless of group, fail to modulate their behavior to optimize outcomes by selecting the risky option more frequently when it was more likely to yield a larger reward. Since no effect of probability was found on risk preference, we collapsed across probabilities for subsequent analyses. As seen in Figure 3A, after correcting for multiple comparisons, posthoc contrasts showed no difference between groups' risk preferences on 100% probability of success; however, when combined across variable probabilities, EtOH-High animals demonstrated significantly higher risk preferences than Control animals (t = −2.95, p = 0.03, Cohen's d = 1.47). Additionally, while Control and EtOH-Low animals became risk averse over repeated exposure to variable probabilities of success (Figure 3B), EtOH-High animals did not, differing from control animals on variable risk sessions 3–7 (Figure 3C; all p≤0.05, all Cohen's d≥1.12).

**Figure 4:**
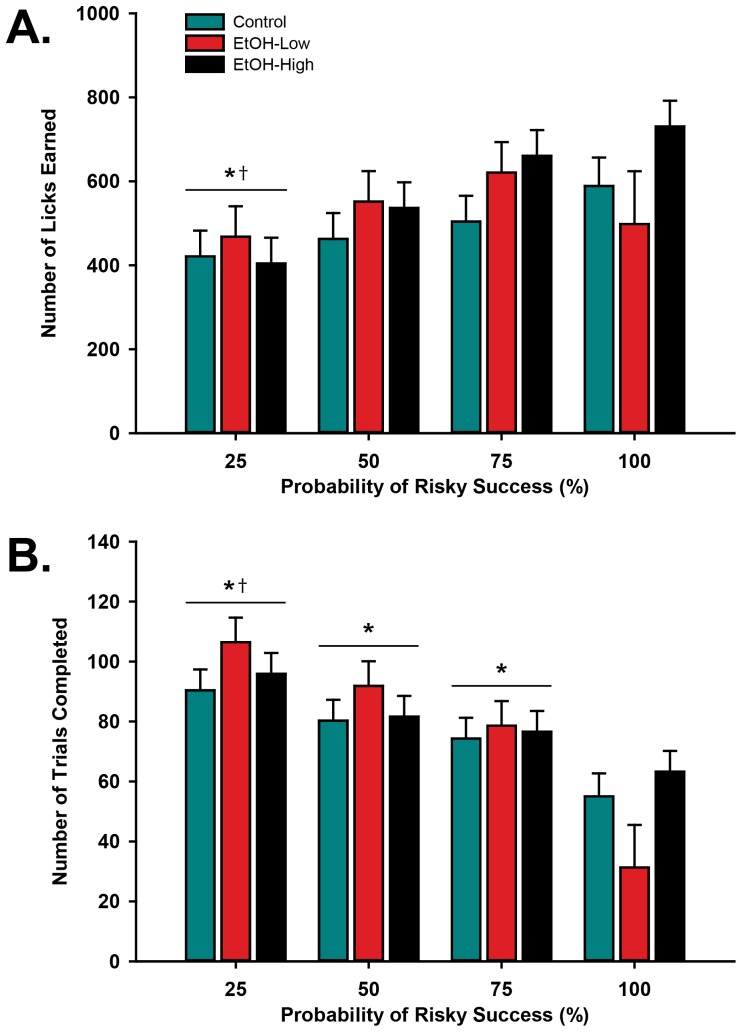
The effects of adolescent alcohol exposure on risk task successfulness. Across all groups, 25% success probability resulted in fewer licks than 100% probability (A); however, this was offset by an increase in the number of trials completed in the variable risk (B), with more trials completed as the probability of success became lower. Note: * denotes difference from 100% (p≤0.05); † denotes difference from 75% (p≤0.05, ††p<0.01).

While risk preference did not differ according to the probability of risky payoff, the number of licks earned (Figure 4A) was affected by the probability of risky payoff [F(3,71) = 4.05, p = 0.013; η^2^ = 0.06], with all groups earning fewer rewards on sessions with 25% probability than sessions with 100% (p = 0.044) and 75% (p = 0.018) probabilities. This effect was offset by all groups completing more trials on the lower probability schedules [F(3,71) = 15.09, p≤0.001; η^2^ = 0.17] (Figure 4B). Posthoc comparisons showed that across groups, animals completed fewer trials on 100% probability sessions than on 25% (p≤0.001), 50% (p≤0.001), and 75% (p = 0.004) probabilities, and fewer trials on 75% probability sessions than on 25% probability (p = 0.006). Combined, these results suggest that when animals earn fewer rewards (lower probability of risky payoff), they increase the number of trials they complete, but do not shift the strategy used.

### Experiment 2: Effects of adolescent alcohol exposure on flexible behavior

**Figure 5:**
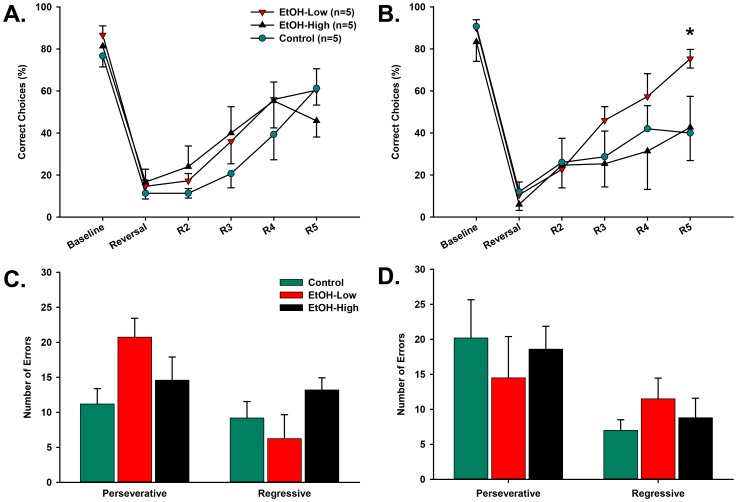
Adolescent acquisition and reversal learning during a spatial discrimination task. Average percent correct for the last day of acquisition (baseline) and the first five days following reversal. There was no difference due to alcohol access following the first reversal (A), nor were there differences in the number or type of errors following the first reversal (C). Adolescent rats also did not differ on performance following the second reversal, except for the last day of testing, when EtOH-Low animals showed a higher percent correct than EtOH-High and Control animals (B), but did not differ in the number or type of errors (D). Note: * denotes difference from Control (p≤0.05).

A reversal learning task was used to assess the effect of ethanol on rats' ability to adapt behavioral choices to changing reward contingencies (Figure 2B). Ethanol exposed (EtOH-High, EtOH-Low) and Control groups acquired the initial discrimination between rewarded and unrewarded ports at similar rates. The average number of days to reach criterion for EtOH-High (7.4±1.2), EtOH-Low (7.2±0.4), and Control (7.4±0.9) groups was not significantly different [F(2,12) = 0.01, p = 0.98]. Across the days spanning the initial reversal (Figure 5A), there was an expected main effect of day [F(11,131) = 26.14, p<0.001]. Posthoc analysis showed that on the first reversal day (‘Reversal’ on P35) there was an expected decline in performance as the contingencies were switched (p<0.001). From P36–39 (R2–R5), all rats learned the new contingencies, demonstrated by an increase in percent correct choices of the new rewarded port over time. However, there was no significant main effect of group across days [F(2,131) = 1.9, p = 0.19], nor were there significant day × group interactions [F(22,131) = 0.93, p = 0.56].

Prior to the second reversal session (Baseline, Figure 5B), there was no difference in performance between groups (p = 0.83). On the second reversal day (‘Reversal’ on P45) there was again a significant decline in overall performance when contingencies were reversed back to the original contingencies (*p*<0.001). Following the second reversal, there were again no significant differences between groups in retention performance except on the last day of testing (P49/R5), when EtOH-Low demonstrated statistically better performance than both EtOH-High (p≤0.05) and Control animals (p≤0.05). The results suggest that ethanol exposure did not affect the ability to flexibly adapt to changing cue-response-outcome relationships.

An analysis of the errors that occurred during both the first and second reversals indicated that the ethanol exposure did not affect the ability to initially inhibit previously rewarded contingencies (perseverative errors) or the ability to maintain the new choice pattern (regressive errors). Specifically, the number of perseverative errors made did not differ between groups [F(2,24) = 2.05, p = 0.15] during the five days following either the initial reversal (P35–39/R2–R5, Figure 5C) or the second reversal (P45–49/R2–R5, Figure 5D). Further, all groups committed a comparable number of regressive errors [F(2,24) = 1.73, p = 0.2] following both the first (Figure 5C) and second reversals (Figure 5D).

## Discussion

Using a translational model of voluntary binge alcohol consumption in adolescents, we assessed the impact of alcohol intake on risk preference and behavioral flexibility during adolescence. During this stage of development, risk preference was related to consumption of ethanol, with high alcohol consuming (EtOH-High) rats preferring large, risky outcomes compared with Control animals, but not low alcohol consuming (EtOH-Low) animals, which showed an intermediate level of risk preference. While adolescent ethanol consumption has been previously shown to elevate risk preference and neural signaling of rewards in adulthood, it was not clear whether the effect of ethanol was due to immediate or longer-term alterations in developing neural circuitry. Using a similar method of gelatin-ethanol exposure in adolescence, Nasrallah and colleagues showed that adult rats adapted their behavior according to reward probability, but that adults who consumed ethanol in adolescence displayed a bias for the larger uncertain rewards over small certain rewards [7], [22], [23]. Gelatin-ethanol exposure during adolescence also supported more rapid acquisition of associations between cues/responses and rewards in both Pavlovian and instrumental tasks in adults, but did not affect rates of extinction [22]. Together, these results suggest that the risk bias exhibited in adulthood is related to an over-learning of reward-associated cues and behaviors, potentially supporting the idea that reward valuation is altered, rather than incentive salience. This regimen of ethanol exposure also altered the relative responses of dopamine in the nucleus accumbens to risky and certain rewards, but not dopamine responses to differently sized rewards [23]. This restriction of altered dopamine signaling to rewards only in the risk task suggests that biases in behavior may relate to a diminished capacity to estimate reward probability.

Like these adults, we found that EtOH-High males tested during adolescence showed an elevated risk preference. Additionally, in our risk task, both the control animals and the EtOH-low animals showed clear decreases in risk-preference over time, while the EtOH-High animals did not, suggesting differential tactics used between groups. It is not clear from the present studies whether the elevated preference in the EtOH-high group stems from differences in reward valuation, incentive salience, or sensitivity to losses in the risk task. There were no reliable differences is preference for the risky over the certain option in the 100% training condition, although behavior for these sessions was more variable than that observed in the reversal learning task. The difference between tasks suggest that reward omission has a strong influence over behavior, but that sensitivity to probabilistic reward omission is modulated by ethanol exposure. While this is consistent with previous findings in adults [22], [23], here, adolescent rats consuming ethanol did not show any differences in acquisition of our instrumental task during adolescence. The current results cannot clearly rule out differences in incentive salience, as we did not directly measure hedonic responses to delivered rewards to quantify whether they correlate with preference for the risky option in adolescents.

The probability schedules used in our task were chosen such that choosing the risky option would have been beneficial over time. The expected values of the risky option outweighed the certain on both the 75% and the 50% schedules (75%: 5 licks vs 11.5; 50% 5 vs 7.5). Thus, while the tactic adopted by the EtOH-High animals cannot be labeled “maladaptive”, it can be labeled abnormal due to its difference from other groups. It is surprising that such a reduction in risk preference exists in Control and EtOH-Low animals, but demonstrates that adolescent behavior is not necessarily dictated by utility functions. In our previous work, adult rats typically displayed either a preference for, or indifference toward, a risky outcome with equal expected value to the certain [30]. Here, we find adolescent rats to show a reduction in risk preference over time in Control and EtOH-Low animals, but not EtOH-High, when omission of reward is introduced into the task. This avoidance of the risky lever persists despite a higher expected value of the risky lever for the 50% and 75% sessions. These differences in choice biases could highlight the transition from adolescent behavioral patterns to adult patterns, which our data would suggest is impaired in EtOH-High animals.

Our adolescent animals did a relatively poor job of matching their behavior to risky reward probability. This lack of behavioral modulation by reward probability could result from reduced task engagement, due to the lack of food deprivation in our model. Adolescent animals were not food-restricted in our paradigm to reduce the potential confounds associated with deprivation in a developing animal. Therefore, motivation to engage in the behavioral task may have been reduced, attenuating the animals' sensitivity to changing probabilities. However, the high number of trials completed by animals may temper this argument. An alternative explanation would be that adolescents adopt a response bias that does not change according to feedback from the outcomes of choices. Indeed, human adolescents have been shown to tolerate ambiguity more than adults, biasing them towards continued engagement in risky tasks and a higher preference for risk in changing contingencies [32]. Given such a potential bias in our animals, it is important to dissociate preference for risk from an inability to flexibly adapt to changing action-outcome contingencies. Here, we showed that in the lowest probability of risky payoff, animals earn the fewest rewards, but complete the most trials. This suggests that instead of changing response strategy (ie. shifting to the certain option), animals instead adapt to the new contingencies by completing more trials. In the risk task, the association of the ‘risky’ option with large reward may have interfered with the rats’ ability to shift responses to the ‘certain’ option, even when it was objectively more valuable (e.g. when the risky option only yielded large reward on 25% of chosen trials). Thus, we also tested the impact of alcohol directly on behavioral flexibility using a reversal learning paradigm. Here, we found that adolescent ethanol exposure did not substantially affect the animals' ability to flexibly adapt behavior to changing reward contingencies. This pattern of results indicates that the overall increases in risk preference observed in high ethanol consumers were not a reflection of the animals' inability or reluctance to adjust behavior in response to changes in associated expected value.

While our studies found no deficits in behavioral flexibility in adolescence, prior studies have shown either deficits [16], [24], [25], [26] or no effect [33] of adolescent alcohol intake on subsequent flexibility in adulthood. While these previously reported effects may be a latent effect of adolescent alcohol intake, it is also possible that task differences contribute to differing assessments of behavioral flexibility. Prior studies showing deficits in behavioral flexibility after adolescent ethanol treatment utilized the Barnes Maze [24] or Morris Water Maze [16], [26] visuospatial reversal learning tasks. Here, we used an operant task in place of a visuospatial task, one that was similar in nature to the risk task, so that comparisons of behavioral performance could be made between tasks. One notable comparison is the differences between the proportion of choices for the ‘correct’ port in the reversal task and the ‘certain’ port in the risk task, both of which yield reward every time they are selected. In the reversal task, in which the alternative (‘incorrect’) port never yields reward, rats quickly and uniformly adopt a strong preference for the ‘correct’. However, in the risk task where the alternative (‘risky’) lever yields a larger payoff on a probabilistic schedule, EtOH-High rats in particular show a greatly elevated preference, even during sessions when reward is omitted on the majority of risky choices. These behavioral results suggest altered representation of reward or associative learning processes that drive behavior toward an option that may be irrationally overvalued. This type of modulation of reward signaling has been observed in adulthood following voluntary adolescent ethanol consumption [23]. Future studies will examine how ethanol consumption alters neural encoding of reward and reward-predictive cues in adolescents.

These two behavioral tasks were specifically designed to overlap in numerous ways, as shown by the overlap of the designs shown in Figure 2. For example, the cues, operant responses, and reward (Ensure) were all matched across tasks. Importantly, the absolute difference between the high and low reward size on the risk task (15 licks versus 5) was the same magnitude as the difference in reward size in the reversal task (10 licks versus 0 licks). The major difference between the tasks was that in the risk task, there was chance the reward would not be omitted. This important difference led to drastically different performance on the two tasks, despite the other similarities. When there is certainty about omission (as in the reversal task), the animals consistently avoided that option. However, in the risk task there is a chance of omission associated with the large reward, yet the animals are not biased against this omission, particularly in EtOH-High animals.

Other methodological considerations may temper the ability to measure robust behavioral differences during adolescence. Many studies investigating the effects of ethanol consumption at this stage of development have relied on non-translational routes of administration (e.g. intragastric infusion, intraperitoneal injection, or inhalation), which provide controlled dosing and enable the achievement of higher BEL's. Here, adolescent rats were allowed to freely consume ethanol in a gelatin solution, which resulted in greater variability in intake, but average consumption values seen in our EtOH-High animals fell slightly below NIAA established definitions of “binge” [34]. Despite the low volumes of EtOH consumed, we still found reliable behavioral differences in the risk task. Voluntary administration reduced potential stress, which may be induced by intragastric infusions or injection and may compound the effects of alcohol during this sensitive period in development to alter future behavior (for review, see [35]). To further minimize stress, we adopted a semi-restricted social context during ethanol consumption, which allowed the animals to interact, but permitted the monitoring of individual consumption levels. Social isolation can have a debilitating effect on a developing animal, including an increase in anxiety-like and depressive-like behaviors [36] and even increased self-administration of ethanol [37]. Although the semi-restricted environment we used here was intended to reduce stress, we did not measure any markers of stress reactivity (behavioral or hormonal), and thus cannot definitively state that our efforts lowered stress. Indeed, it is even possible that the altered environment may have increased stress above levels seen during total isolation. Future studies will explore the impact of this housing environment on acute and long-term stress.

While the daily consumption levels reported here are lower than forced models, the gain in translational relevance cannot be understated. Our EtOH-High animals consumed more EtOH gelatin per hour than previously reported in voluntary gelatin access models, which provide continuous, 24 hour access [7], [23]. Furthermore, the one-hour access period used here more accurately models other models of binge consumption, like “drinking in the dark”, and permits more accurate estimations of BELs without disruptions of the exposure period. Estimation of BELs is pivotal to our understanding of the effects of our access model on neuropharmacology.

The results shown here relate the impact of adolescent alcohol on risk preference only in male rats. Reports of sex differences in impulsive behavior have been mixed (for review see [38]), but may play a role both in the initial acquisition of alcohol consumption and the transition to continued abuse. While males and females show similar rates of alcohol abuse during adolescence [1], it is possible that alcohol intake during adolescence affects each sex differently, and may interact with latent differences in risk preference. Future studies should explore whether sex differences exist in the short and long term effects of adolescent alcohol intake.

In summary, repeated voluntary ethanol consumption during adolescence has no effect on reversal learning in adolescence, but can increase risk preference in high consuming adolescents. The bias towards large risky rewards displayed by rats exposed to alcohol during adolescence suggests that they may over-value the large reward following exposure to ethanol. Additionally, we found that the negative effects of ethanol begin during the onset of ethanol consumption. Future studies using this model should clarify the causal link between ethanol consumption and preference for risky situations. Ultimately, such results may contribute to our understanding of the relationship between ethanol consumption during adolescence and maladaptive decision-making throughout the lifespan.
